# Understanding the clinical and environmental drivers of antifungal resistance in the One Health context

**DOI:** 10.1099/mic.0.001512

**Published:** 2024-10-30

**Authors:** Catrin C. Williams, Jack B. Gregory, Jane Usher

**Affiliations:** 1 Medical Research Council Centre for Medical Mycology, University of Exeter, Exeter, UK

**Keywords:** AFR, AMR, *Aspergillus*, azoles, *Candida*, One Health, resistance

## Abstract

Antifungal drugs have had a tremendous impact on human health and the yields of crops. However, in recent years, due to usage both in a health setting and in agriculture, there has been a rapid emergence of antifungal drug resistance that has outpaced novel compound discovery. It is now globally recognized that new strategies to tackle fungal infection are urgently needed, with such approaches requiring the cooperation of both sectors and the development of robust antifungal stewardship rationales. In this review, we examine the current antifungal regimes in clinical and agricultural settings, focusing on two pathogens of importance, *Candida auris* and *Aspergillus fumigatus,* examining their drivers of antifungal resistance, the impact of dual-use azoles and the impact agricultural practices have on driving the emergence of resistance. Finally, we postulate that a One Health approach could offer a viable alternative to prolonging the efficacy of current antifungal agents.

## Introduction

In recent years, globalization has increased with improved worldwide travel and trade, resulting in the introduction of antifungal-resistant fungal pathogens into novel environments globally [1,2]. Maintaining an effective antifungal arsenal is critical to protecting both human health and crop security [3,4]. To achieve this, an integrated One Health approach, which considers antifungal resistance (AFR) drivers both in clinical and environmental settings, is required [5]. There are five main strategies which could be implemented to tackle the increasing AFR: development of novel antifungals, implementation of antifungal stewardship (AFS), integrated disease management and improved diagnostics and surveillance [1,6, 7]. In this review, we will focus on the usage of antifungals and how this has impacted the emergence of cross-AFR in fungal pathogens.

**Fig. 1.**
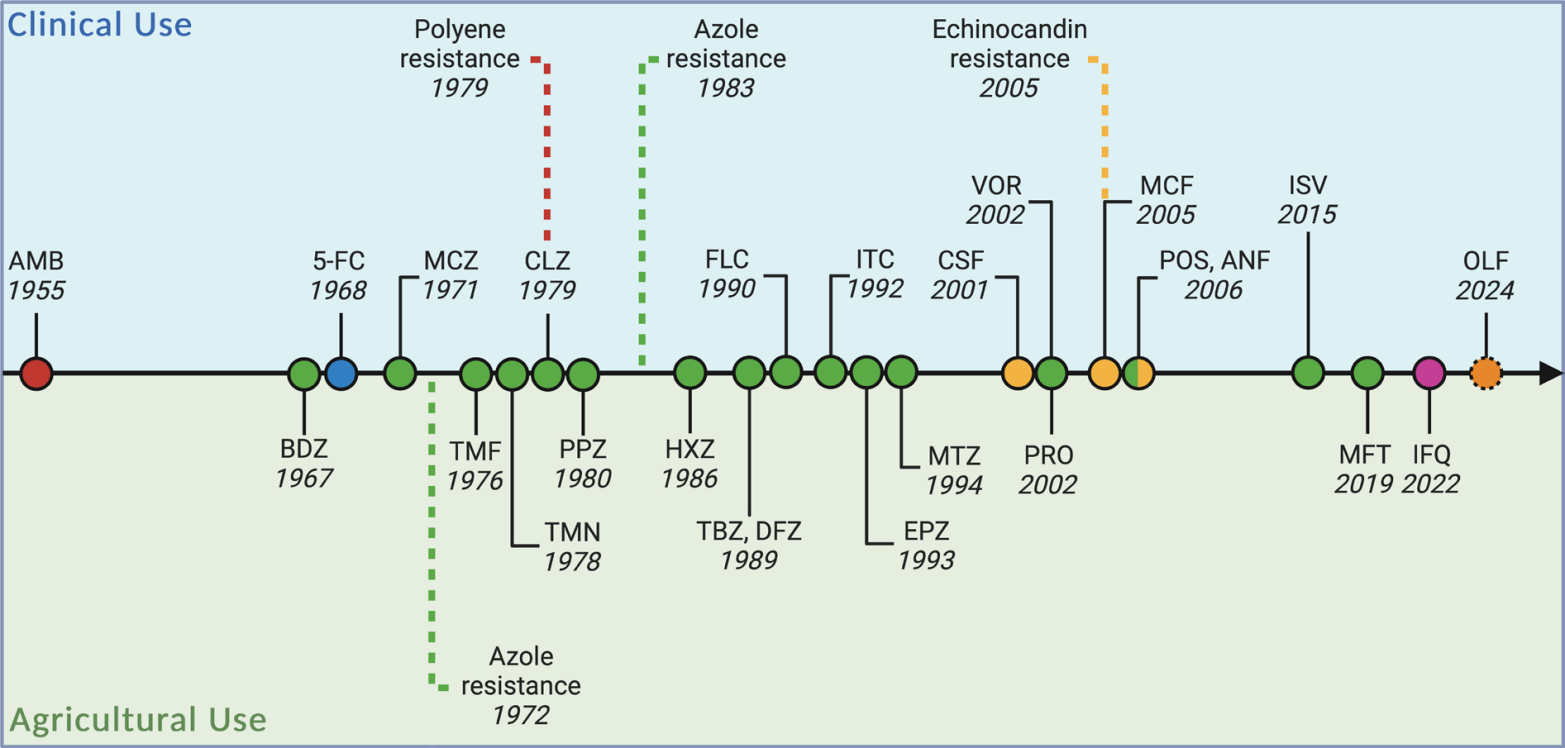
Antifungal drug development and resistance emergence timeline, separating antifungals into clinical or agricultural use. Coloured points refer to their antifungal drug class: polyenes (red), pyrimidine analogues (blue), azoles (green), echinocandins (yellow), quinolines (pink) and orotomides (orange). Dashed lines refer to the first reported case of resistance to the drug class either in the clinical or agricultural settings [199,202]. Point borders refer to drug development status: approved/in use (full/closed) and in development (dotted/broken). AMB, amphotericin B; BDZ, benzimidazole; 5-FC, 5-flucytosine; MCZ, miconazole; TMF, triadimefon; TMN, triadimenol; CLZ, clotrimaconazole; PPZ, propiconazole (expired regulatory status); HXZ, hexaconazole (expired regulatory status); TBZ, tebuconazole; DFZ, difenoconazole; FLC, fluconazole; ITC, itraconazole; EPZ, epoxiconazole; MTZ, metconazole; CSF, caspofungin; VOR, voriconazole; PRO, prothioconazole; MCF, micafungin; POS, posaconazole; ANF, anidulafungin; ISV, isavuconazole; MFT, mefentrifluconazole; IFQ, ipflufenoquin; OLF, olorofim. Created with BioRender.com.

There is an inherent lack of effective antifungals which are licensed for use clinically and agriculturally (Fig. 1). This is further exacerbated by the decreasing number of antifungals which are licensed within agriculture due to associated ecotoxicology issues [8,10]. Current antifungal treatments available clinically are often inadequate, with mortality rates remaining high [6], especially with the emergence of novel multi-drug-resistant (MDR) fungal pathogens such as *Candida auris* [11], as discussed below. Therefore, there is an urgent need to develop novel therapeutic antifungals. Ideally, novel antifungals would be fungicidal rather than fungistatic (Table 1), allowing for increased efficacy and thus reducing the emergence of resistance [12]. If novel antifungals could reach the infection site and be metabolized efficiently, this would minimize the antifungal compounds being excreted into environmental reservoirs [6,13]. The development of novel therapeutic targets for fungal infections is challenging because many fungal genes share significant homology with human genes, particularly in essential cellular pathways. This genetic similarity complicates drug development, as targeting fungal enzymes or proteins often risks affecting human counterparts, leading to potential toxicity or side effects. Consequently, identifying fungal-specific targets that minimize harm to human cells requires careful and detailed molecular differentiation, making the discovery process both time-consuming and complex [14]. Therefore, developing novel antifungals which fulfil these ideal criteria remains challenging. However, it is essential to recognize that our understanding of AFR dynamics is continually evolving, and ongoing research in this emerging field may bring forth new insights that refine our perspectives on the interplay between agricultural practices and clinical outcomes [15,16].

**Table 1.** Definitions used in this manuscript

Term	Abbreviation	Definition	Section
Antifungal resistance	AFR	AFR refers to a heritable change within a fungus which confers reduced antifungal susceptibility	Introduction
Fungicidal	**/**	A drug which results in the death of a fungus	Introduction
Fungistatic	**/**	A drug which inhibits fungal growth	Regimes and resistance in a clinical setting
Antimicrobial resistance	AMR	Reduced susceptibility of fungi, bacteria, viruses and parasites to previously effective drugs	Regimes and resistance in a clinical setting
Multi-drug resistance	MDR	Resistance of a fungus to more than one antifungal class.	Drivers of AFR in a clinical setting
Minimum inhibitory concentration	MIC	The lowest antimicrobial concentration which inhibits visible microbial growth following incubation overnight	*C. auris* – the new growing fungal threat
Single nucleotide polymorphisms	SNPs	A change in a single nucleotide within a gene compared to its wild-type	*C. auris* – the new growing fungal threat
Acquired resistance	/	The development of AFR following exposure to antifungal pressure.	Dual use of antifungals within the environment and clinic
Minimal selective concentration	MSC	The lowest antimicrobial concentration which positively selects for resistance.	Crosstalk between the environment and clinic
Environmental risk assessments	ERA	A method to measure the probability that a chemical compound will cause unwanted ecological effects.	Crosstalk between the environment and clinic
Intrinsic resistance	/	Inherent resistance of a fungus to antifungals without prior antifungal exposure.	Other anthropogenic factors driving resistance: globalization and climate change
Monoculture	/	Solely growing a single crop species at a time.	Agricultural practices driving AFR

## Regimes and resistance in a clinical setting

Fungal species cause a range of illnesses and disease severity, resulting in approximately 2.5–3.8 million annual deaths globally, compared to the 1.6 million deaths per year caused by tuberculosis (TB) and the 619 000 deaths per year caused by malaria [17,20]. Whilst not all fungal diseases lead to death, other lifestyle-affecting diseases can also be caused: minor superficial mycoses, e.g. pityriasis versicolour, caused by *Malassezia globosa* and *M. furfur* affecting 2–8% of the global population, and cutaneous and subcutaneous mycoses, e.g. onychomycosis, caused by the dermatophyte genera of *Trichophyton, Epidermophyton* and *Microsporum,* with an incidence of 5–25% of the global population [21,22]. Mucosal fungal infections are mostly caused by the commensal species of the *Candida* genus, with vaginal, oesophageal, oropharyngeal and urinary tract candidiasis being common in immunocompromised patients. Globally, 372 million women are affected by recurrent vulvovaginal candidiasis over their lifetime, with an estimated 20 million extra cases by 2030, resulting in an associated socio-economic burden of US$14.39 billion [23]. Taken in their entirety, fungal infections are estimated to affect 1.7 billion people each year, with 150 million cases being severe and life-threatening [24,25]. The number of global fungal infections is rising due to the increase of at-risk populations, such as the elderly, critically ill, organ transplant recipients and immunocompromised patients [26,27]. Whilst the estimates provided offer valuable insights into the extensive impact of fungal infections, it is crucial to recognize the need for more robust quantitative epidemiological data. Unlike well-established diseases such as TB and malaria, the lack of precise figures for most fungal infections underscores the imperative to invest in comprehensive research efforts. Obtaining accurate data not only refines our understanding of the true burden of fungal diseases but also presents an opportunity to make a compelling case for increased research funding, ultimately contributing to more effective public health strategies.

Within the clinic, there has historically only been a limited number of available drug classes to combat invasive fungal diseases, which target distinct fungal metabolic pathways. These drug classes are fluoropyrimidines, polyenes, azoles and echinocandins [17]. However, only polyenes, azoles and echinocandins can effectively treat invasive fungal infections [28]. Antifungal drugs are then further classified as either fungicidal or fungistatic (Table 1) [29]. Fluoropyrimidines, of which only 5-fluorocytosine and 5-fluorouracil are used in the clinic, act by disrupting RNA and DNA biosynthesis by replacing UTP (Uridine triphosphate) during transcription, inhibiting protein synthesis and inhibiting thymidylate synthase during DNA replication [30,31]. These are fungistatic against *Candida* spp., in combination with azoles and *Cryptococcus* spp. in combination with polyenes [29]. Whilst over 200 molecules belong to the polyenes, only three are used in the clinic due to toxicities: amphotericin B (AMB), nystatin and natamycin [29]. By binding to ergosterol on the fungal membrane, the permeability of the cell membrane is affected and pores form due to the chemicals’ amphiphilic structure [29,32]. Polyenes can be used against a wide range of fungal species and have fungicidal activity against, but not limited to, *Aspergillus*, *Cryptococcus* and *Candida* spp. [33,34]. The echinocandins, including caspofungin (CSF), micafungin and anidulafungin [35,37], are inhibitors of β(1-3)-glucan synthase and disrupt the polymerization and biosynthesis of β(1-3)-glucan, affecting cell wall integrity and rigidity [38]. Echinocandins are fungicidal against *Candida* spp. and fungistatic against *Aspergillus* spp., yet have no activity against *Cryptococcus* spp. [39,41]. Azoles are the most used antifungals in the clinic and are separated into two groups: the imidazoles, which contain two nitrogen atoms in the azole ring, and the triazoles, which contain three nitrogen atoms [42]. All azole drugs target lanosterol 14α-demethylase, a key enzyme in the ergosterol biosynthesis pathway, resulting in the synthesis of toxic compounds that cannot replace ergosterol [43]. Most azoles are active against *Candida*, *Aspergillus* and *Cryptococcus* spp., alongside other fungal species [44]. Newer generation triazoles such as voriconazole (VOR) and posaconazole (POS) were Food and Drug Administration-approved in the early 2000; fungal isolates resistant to classical triazoles such as fluconazole (FLC) and itraconazole (ITC) are showing cross-resistance to the newer generation triazoles [45].

While these antifungal drugs have played a critical role in clinical practice, their continued use has contributed to the emergence of antimicrobial resistance (AMR) among fungal pathogens, with multiple studies identifying fungal species resistant to one and/or more classes of antifungals, also being linked to therapeutic failure [46,48]. Notably triazole-resistant *Aspergillus fumigatus* and MDR *C. auris* are examples of worldwide AMR emergence within fungal species [49,50].

## Drivers of AFR in a clinical setting

In clinical settings, AFR spreads through inadequate infection control practices, such as poor hand hygiene and improper sterilization of medical devices, which allow resistant fungi to persist and be transmitted between patients. Overuse or misuse of antifungal medications, often due to empirical treatment in the absence of accurate diagnostics, accelerates the selection of resistant strains. Medical devices like catheters and endoscopes can harbour biofilms of resistant fungi, which are difficult to eliminate with standard cleaning protocols. Additionally, cross-contamination in high-risk areas, such as ICUs, (Intensive Care Units), further facilitates the spread of resistant fungal infections among immunocompromised patients.

Antifungals are prescribed by a wide spectrum of specialities to treat fungal infections [51,52]. The challenges associated with accurate and timely diagnosis of fungal infections can lead to empirical antifungal prescription, often resulting in the emergence of resistance [27]. This has been shown within the Michallon University Hospital that correlated historic increased antifungal prescription with subsequent increased ICU *Candida* spp. drug resistance profiles for all four antifungal drug classes [53]. Misuse and overprescription of combined steroid, antifungal and antibacterial creams are also driving the emergence of resistance in the previously mentioned dermatophyte genera, notably *Trichophyton* with cases showing MICs >32 mg l^−1^ against terbinafine [54,55]. The routine use of chemotherapies, immunosuppressive drugs, broad-spectrum antimicrobials and monoclonal antibodies (mAbs) with immunological properties results in immunocompromised windows, allowing for opportunistic invasion by fungal species. In immunocompetent individuals, fungal disease is often prevented due to the intact immune system and high body temperature, resulting in low fungal disease rates amongst healthy populations [56].

Medical devices such as catheters are a common source of hospital-acquired infections, accounting for up to 80% of all bloodstream, urinary tract and pneumonia-related infections [57]. With at least 18 million gastrointestinal endoscopies conducted in the United States of America each year, the scope and scale of surgical tools and medical devices contacting patients’ sterile tissue can start to be appreciated [58]. Whilst established treatment and sterilization methods effectively kill free-living fungi, the ability of specific fungal species to form persistent biofilms on these surfaces poses a risk, with many studies showing *Candida albicans* biofilms to be 30–2000-fold more resistant to azoles and polyenes [59]. Fungal biofilms can also form on indwelling medical devices, with *Candida* spp. forming biofilms on a range of synthetic materials [60,64]. As a result, biofilm-related infections often require either the removal of the infected device or higher doses of antifungals, inadvertently promoting the emergence of AMR [63].

## *C*. *auris* – the emerging fungal threat

First identified in Japan in 2009, *C. auris* is an example of an intrinsically MDR fungus, which emerged independently on three different continents [11,65, 66]. *C. auris* is intrinsically FLC resistant and has displayed cross-azole, polyene and echinocandin resistance [66]. A recent *in vitro* experiment showed that the three passages in the presence of FLC resulted in genomic and phenotypic changes, karyotype alterations, aneuploidy and the acquisition of point mutations, all of which led to increases in MIC values across 17 clinical isolates of *C. auris* [67]. Of concern was the fact that the observed resistance was stable in the absence of the drug, suggesting that there was no fitness cost associated with resistance.

**Fig. 2.**
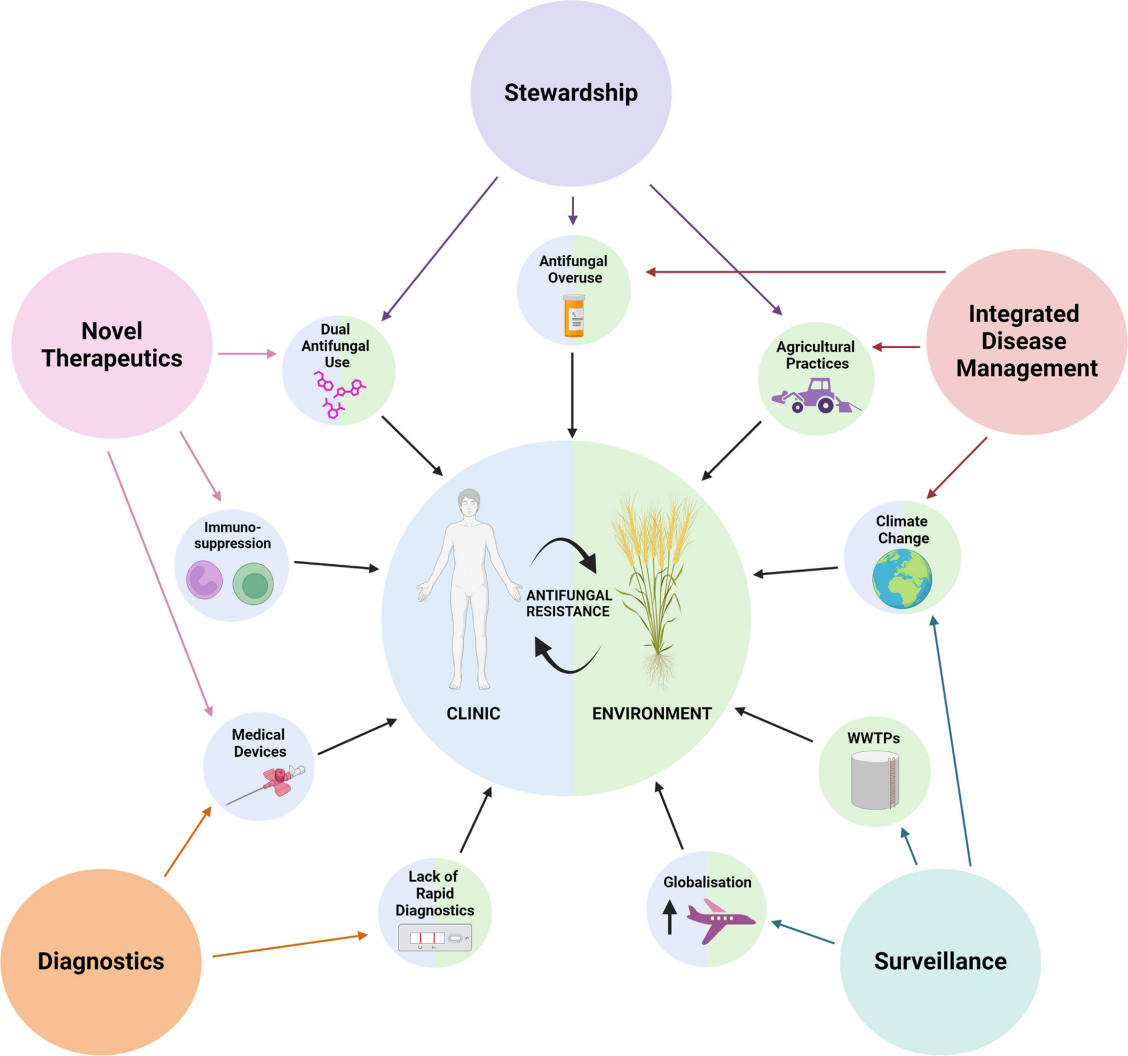
The One Health drivers of AFR. Drivers in solid blue represent clinical drivers of resistance alone. Indwelling medical devices allow for increased biofilm formation, which is associated with resistant and recurrent infections. Increasing immunosuppression due to medical advances increases the population of individuals at risk of a fungal infection. Additionally, their impaired immune system can result in prolonged infection and, therefore, long-course treatment, which creates strong selection pressures for resistance. Drivers of environmental resistance alone are represented in solid green. Wastewater treatment plants (WWTPs) often cannot efficiently clear antifungals from water due to long antifungal half-lives; this introduces antifungal residues into the environment, which creates an indirect selection pressure. Agricultural practices such as monocultures, composting, sludge application, etc. are all practices that increase the selection pressure for AFR. Drivers of AFR in both the clinic and environment are represented in half-blue and half-green. These include dual antifungal use in the clinic and environment (causing increased selection pressures), climate change (increasing fungal thermotolerance), increased globalization (introduction of pathogens to novel environments), overuse of antifungals (increased selection pressures) and lack of rapid diagnostics (delays in selection of effective treatment). The five main strategies to tackle increasing AFR, as proposed by this review, are represented in the larger circles on the outermost edges of the figure. These strategies include increased AFS (in purple), development of novel therapeutics (in pink), improved diagnostics (in orange), increased surveillance (in teal) and integrated disease management (in red). Colour-coordinated arrows indicate which main strategies we propose could decrease the contribution of a particular driver to increasing AFR. Created with BioRender.com.

In addition, *C. auris* has multiple mechanisms of resistance against FLC, such as point mutations and overexpression of *ERG11*, which encodes for the enzyme lanosterol 14α-demethylase that mediates the ergosterol biosynthesis pathway. Three hot spot regions exist within *ERG11*, aas 105–165, 266–287 and 405–488, and these have been supported by various studies identifying SNPs within these regions leading to increased MICs against azoles [29,68]. ATP-binding cassette and major facilitator superfamily are the two major families of efflux pumps involved in AFR in *C. auris*. Overexpression of these efflux pumps leads to major resistance to the azoles, with recent studies showing that the deletion of a gene homologous to *C. albicans CDR1* in *C. auris* increased azole susceptibility of resistant strains 64–128-fold [69,71]. Mutations in transcription factors also result in increased FLC resistance; a single mutation in the zinc cluster transcription factor *UPC2* has been shown to correlate with a twofold increase in FLC MIC_50_ [72]. *C. auris* also highlights the previously mentioned issue of ineffective decontamination procedures, with the species able to adhere to a variety of nosocomial surfaces and survive standard disinfection procedures [73]. The resistance properties of *C. auris* and its rapid global spread highlight the clinical drivers of AMR within fungal species (Fig. 2).

## Antifungal usage in agricultural environment

Alongside causing a significant clinical burden, fungal pathogens are a leading threat to crop security [4], with approximately 20% of global perennial yield losses and a further 10% of post-harvest losses attributed to fungal pathogens [1]. Fungal pathogens, therefore, represent a significant threat to economic security alongside crop security. This is exemplified by *Zymoseptoria tritici,* the causal agent of Septoria tritici blotch (STB) in wheat (*Triticum aestivum*) [74]. Wheat is a staple crop that is responsible for 18.3% of the global human calorie intake [75]. In the UK alone, STB causes £240 million in crop losses [76]. Therefore, to control this threat, antifungals are utilized widely within agriculture [77].

Azoles represent the most frequently utilized class of antifungals agriculturally, as they are inexpensive and broad-spectrum [78,79]. Azoles are classified as demethylase inhibitor fungicides, examples of which include difenoconazole (DFZ), epoxiconazole (EPZ), propiconazole (PPZ) and tebuconazole (TBZ) [80]. Azoles inhibit the enzyme CYP51, also known as lanosterol 14α-demethylase [81], which plays a role in sterol biosynthesis. In fungi, CYP51 [78,82] is often encoded by a single *CYP51* gene. However, in some filamentous fungi, particularly ascomycetes, multiple paralogous *cyp51* genes can be present [81]. Azoles were first used agriculturally in the late 1970s (Fig. 1) [82]. Currently, approximately 30 000 metric tons of azoles are utilized within both the EU and China [77,79]. The vast use of azoles agriculturally has introduced a strong selection pressure for azole resistance within crop fungal pathogens [1]. Similar to human fungal pathogens, crop pathogens display three main azole resistance mechanisms: mutations within the *CYP51*, overexpression of *CYP51* or overexpression of efflux pumps [78,83,85].

Over the past few decades, azoles have been the cornerstone of *Z. tritici* control [86], particularly in Europe, where the majority of *Z. tritici* populations display strobilurin resistance [83,87]. This increased resistance of *Z. tritici* populations has been predominantly attributed to nonsynonymous mutations within *CYP51*; however, similar to other fungal pathogens, both overexpressions of *CYP51* and efflux pumps can contribute to azole resistance [86,88, 89]. This is often mitigated by increasing the dose applied to crops; however, this does not represent a feasible solution to azole resistance [90,91]. Additionally, there are fewer fungicides licensed for the treatment of *Z. tritici* [86]. These banned antifungals include the azoles PPZ and EPZ [86]. Therefore, it is imperative that agricultural treatment regimens are stringent and well-informed to avoid unnecessary antifungal usage.

This is exemplified by the Australian government, enacting one of the most robust AMR programmes globally [92]. Currently, 98% of the registered fungicides within Australia are azoles [93,94]. Azoles only began to be utilized routinely to control plant fungal pathogens in 2002 [94]. However, by 2010, *Z. tritici* became a serious threat to wheat crops, resulting in farmers applying azoles ~2–3 times per crop in Victoria and Tasmania [94,95]. By 2011, azole-resistant *Z. tritici* populations were observed in these regions [94,95]. McDonald *et al.* performed whole-genome resequencing of *Z. tritici* populations within Victoria and Tasmania pre- and post-introduction of azoles agriculturally [94]. As Tasmania is geographically isolated, this analysis provided unique insights into the evolution of azole resistance within a genetically distinct *Z. tritici* population [94]. Indeed, Tasmanian *Z. tritici* populations possessed complex azole-resistance conferring mutations within *CYP51* only ~1–2 years after *Z. tritici* populations were first established within Tasmania [94]. Seven nonsynonymous mutations were observed in the *CYP51* gene within Tasmanian *Z. tritici* populations [94]. This combination of mutations is referred to as isoform 11 and took ~30 years to evolve within European populations [88,94, 96]. Notably, the maximum triazole dose utilized within Australia is ~50% less than the triazole dose utilized in the UK [90,94]. However, this selection pressure from agricultural azole use, within 9 years of the introduction of azoles, was sufficient to establish azole resistance within Tasmanian *Z. tritici* populations [94,95], emphasizing the need to carefully consider which antifungals are applied and at which doses.

Although *Z. tritici* represents the leading threat to wheat crops [76], wheat powdery mildew caused by *Blumeria graminis* f. sp. *tritici* also represents a major threat to wheat crops [78,97]. Without the use of fungicides, powdery mildew can reduce grain yield by ~30% [98,99]. In Europe, first-generation azoles such as triadimefon, triadimenol and PPZ were introduced to control powdery mildew epidemics in the late 1970s and 1980s (Fig. 1) [98,100]. These fungicides were applied intensively, thus introducing a strong selection pressure for azole resistance [98,100]. Subsequently, European *B. graminis* f. sp. *tritici* populations became decreasingly sensitive to azoles [98,100, 101]. The only *B. graminis* f. sp. *tritici* resistance mechanism documented to date is a Y136F mutation within *CYP51* [78,98, 102]. Notably, Czech and British isolates were often cross-resistant to multiple azoles [98,103]. Ultimately, this led to a move away from the use of azoles to control *B. graminis* f. sp. *tritici* within Europe in favour of more effective fungicides [98]. However, azoles are still routinely utilized to control powdery mildew infections in other regions, such as the USA [98]. A study by Meyers and Arellano evaluated the susceptibility of the US *B. graminis* f. sp. *tritici* populations to first- and second-generation azoles [98]. This study demonstrated that there were multiple *B. graminis* f. sp. *tritici* subpopulations within the USA, varying by region, and possessed a range of azole sensitivities [98]. This varied susceptibility profile is likely influenced by differences in azole applications between states [98]. It was observed that resistance rates to the second-generation azole prothioconazole (PRO) were similar to the first-generation azole TBZ (Fig. 1), within American *B. graminis* f. sp. *tritici* populations [98]. The authors hypothesize that the use of TBZ over decades primed *B. graminis* f. sp. *tritici* populations for PRO resistance [98]. No fitness cost was associated with either PRO or TBZ resistance within isolates [98]. When compared to European *B. graminis* f. sp. *tritici* isolates from the 1980s, US isolates are much more sensitive to azoles, likely due to application levels being much lower in the US [98]. Increased surveillance of isolates would allow for early alteration of control measures, e.g. the introduction of rotation of fungicide classes and the introduction of combination fungicides [98]. Preventing *B. graminis* f. sp. *tritici* resistance is crucial, as it is currently in the highest risk category for fungicide resistance in the FRAC (Fungicide Resistance Action Group) guidelines, having evolved resistance to six fungicide classes within 2–5 years of the first introduction of the classes [98,104].

Understanding how resistance emerges is crucial for preventing its development in plant pathogens. The causal agent of rice blast is *Magnaporthe oryzae* [105,107]. Rice (*Oryza sativa*) is a staple crop, used for feeding >50% of the global population [105,108]. *M. oryzae* is considered one of the most significant plant pathogens globally due to its potential to cause failure of an essential crop [105,109, 110]. Fungicides play an essential role in the control of rice blast and are utilized to prevent infection of newly germinated seedlings alongside mature leaves [105,111, 112]. Azoles are commonly utilized to prevent *M. oryzae* infections due to their efficacy [113,115]. Currently, there have been no documented reports of azole-resistant *M. oryzae* agricultural isolates [113]. *In vitro* evolution experiments have shown this is likely due to the heavy fitness cost associated with azole resistance due to nonsynonymous mutations within *CYP51* [113]. *M. oryzae* isolates which were EPZ-resistant displayed cross-resistance to prochlorazole and DFZ [113]. However, the growth rate, sporulation and aggressiveness of resistant isolates were significantly decreased compared to susceptible isolates [113]. Due to this fitness cost, azoles remain an effective tool to control rice blast. Therefore, understanding how other plant pathogens have become azole-resistant and altering agricultural practices to reduce the selection pressure for *M. oryzae* azole resistance is crucial for future crop security.

## Dual use of antifungals within the environment and clinic

It has been proposed that the dual use of antifungals in the environment and clinic acts as a driver of AFR, which is particularly relevant for azoles (Fig. 2) [79,116]. The use of agricultural azoles is hypothesized to create a strong selection pressure for AFR in opportunistic human fungal pathogens alongside crop pathogens [117]. This is largely due to the shared structural similarities with clinical triazoles [isavuconazole (ISV), ITC, POS and VOR] [80]. Consistently, *in vitro* evolution experiments show that the evolution of resistance to agricultural azoles can mediate cross-resistance to clinical azoles [118,119]. This phenomenon has been investigated in *A. fumigatus*, a saprophytic fungus, which resides within the soil ubiquitously throughout the environment [120]. *A. fumigatus* is an opportunistic pathogen causing a range of fungal diseases both in patients and other species within the *Aspergillus* genus, such as *A. flavus,* which is known to cause disease in plants [120]. *A. fumigatus* has become increasingly azole-resistant, reducing the therapeutic options for invasive fungal infections by ~33% [28].

The highly plastic nature of fungal genomes allows fungal pathogens to produce variants possessing potentially beneficial mutations, which can be selected for antifungal stress-acquired resistance [121,122]. *A. fumigatus* possesses two paralogous genes, *cyp51A* and *cyp51B,* which encode CYP51 [123]. Nonsynonymous mutations within *cyp51A* or *cyp51B* can cause structural alterations of CYP51, reducing the binding affinity of azoles, thus decreasing their efficacy [124]. Overexpression of *cyp51A/cyp51B* confers azole resistance [125], commonly arising from alterations within the *cyp51A* promoter region caused by TRs or transposable elements (TEs) [125,126]. Azole-resistant *A. fumigatus* strains can also upregulate efflux pumps ~5–30-fold [127], decreasing intracellular azole accumulation [128]. Crucially, there have been increasing incidences of azole-resistant *A. fumigatus* infections within azole-naive patients [125]. Snelders *et al.* first proposed that the agricultural use of azoles may be driving the emergence of resistance to clinical azoles within *A. fumigatus* [129].

It is widely presumed that the dual use of azoles within the clinic and environment contributes to the emerging resistance, especially within *A. fumigatus* [7,130, 131]. However, this remains a highly debated topic [7,130, 131]. It is crucial to determine whether the widespread use of agricultural azoles is contributing to the emergence of azole-resistant *A. fumigatus*, as this could help guide agricultural practices to slow the development of resistance. Azole-resistant *A. fumigatus* strains isolated from the clinic and environment frequently possess different resistance mechanisms [80]. Isolates from patients undergoing long-term azole treatment often possess single nonsynonymous mutations in *cyp51A* in positions G54, P216, M220 and G448 [80,132, 133]. In particular, nonsynonymous mutations at position G54 are associated with long-term ITC treatment [80,134]. It has been argued that this resistance mechanism may have originated in the environment, as a number of agricultural isolates possessing resistance-conferring mutations at position G54 have been identified in geographically distinct regions, such as Europe, China and Tanzania [135,137]. However, a recent study, which assayed numerous clinical isolates containing a G54 substitution, showed that although isolates were highly resistant to long-tailed clinical azoles (e.g. ITC and POS), they remained susceptible to short-tailed clinical azoles (e.g. VOR and ISV) and agricultural azoles [80], suggesting these clinical isolates had not acquired their resistance mechanism due to agricultural azole exposure. However, the same study showed that the use of the agricultural triazole metoconazole selects for nonsynonymous mutations at position G54 within *A. fumigatus* [80], demonstrating how determining whether agricultural azole use is directly driving the azole resistance within the clinic remains difficult.

Specific mutations in *A. fumigatus* (and other fungi) are often selected for, particularly in response to environmental pressures, such as exposure to antifungal agents [117,119, 125, 130, 138,141]. Mutations such as 34 bp tandem repeat (TR_34_)/L98H and TR46/Y121F/T298A in the *cyp51A* gene confer resistance to azole antifungals [142,143]. While the primary driver of these mutations appears to be the selective pressure exerted by azole use [144,146] (both in clinical and agricultural settings), there is some debate as to whether the selection mechanisms are solely related to antifungal function or if other physiological roles of the protein in the ecosystem contribute to this selection [7,80, 125]. The selection of mutations like TR_34_/L98H in *A. fumigatus* is primarily driven by environmental azole exposure rather than the protein’s role in the ecosystem. These mutations enhance the pathogen’s survival in environments with azoles, and while they might not directly relate to the natural physiological function of the protein, they don't impose a significant fitness disadvantage, which allows these resistant strains to proliferate in various environments, including clinical settings.

There are, however, a number of studies which suggest that *A. fumigatus* is evolving azole resistance within the environment, complicating patient outcomes. A notable example is a TR_34_ within the promoter region of *cyp51A* in combination with an L98H substitution (TR_34_/L98H), which emerged in the Netherlands. Strains with this mutation overexpress *cyp51A* and have point mutations that alter the binding affinity of azoles to *cyp51A*. The TR_34_/L98H mutation has subsequently emerged globally and has become the most common azole resistance mechanism within environmental *A. fumigatus* isolates. This TR_34_/L98H mutation is widely considered a signature mechanism of environmentally acquired resistance due to the use of agricultural azoles. Yet, this mutation confers cross-resistance to ITC and VOR [142,147]. In the Netherlands, 50–71% of azole-resistant aspergillosis cases occur in azole-naive patients, likely due to environmental acquisition of resistance. Similar trends have been observed with the TR_46_/Y121F/T298A mutation, which is also associated with agricultural azole use and cross-resistance to clinical azoles [80,148,151]. Interestingly, *A. fumigatus* strains possessing the TR_34_/L98H and TR_46_/Y121F/T298H mutations have been isolated from azole-naive patients in Denmark, even though environmental isolates from the same period did not possess these mutations [148]. A recent study by Rhodes et al. demonstrated that the clinical azole-resistant *A. fumigatus* isolates were genetically indistinguishable from azole-resistant isolates recovered from the environment [125]. This study suggests that resistance mechanisms gained in the environment due to agricultural azole use do not lead to decreased fitness or virulence of *A. fumigatus in vivo*. This further supports the hypothesis that the use of agricultural azoles is driving the emergence of azole-resistant *A. fumigatus* isolates, which can infect patients and complicate clinical outcomes [125].

Currently, azoles are the first-line therapeutic option to treat invasive aspergillosis infections [152]. However, resistance rates in some hospitals have reached >20%, and without alternative effective treatments, the mortality rates reach ~90% [153,155]. The novel antifungal, olorofim (OLF), is currently in clinical trials and is highly effective against azole-resistant *A. fumigatus* (Fig. 1) [156,157]. OLF belongs to the novel antifungal class, the orotomides [156,158], with a mode of action that inhibits the enzyme dihydroorotate dehydrogenase (DHODH) involved in pyrimidine biosynthesis [156,158]. The novel fungicide ipflufenoquin (IFQ) (commercially named Kinoprol) has recently been approved for use agriculturally in the US and is being considered for use in numerous other countries (Fig. 1) [154,159]. Like OLF, IFQ is a DHODH inhibitor; therefore, there are widespread concerns that IFQ use agriculturally may drive resistance to OLF within the clinic [154,159,162]. A recent study highlighted potential cross-resistance to both antifungals and demonstrated IFQ was active against *A. fumigatus* at a lower dose than the agricultural dose [154]. Ultimately, this study demonstrated that IFQ use can drive cross-resistance to OLF [154]. It is imperative to understand how dual use of antifungals can drive the emergence of resistance to prevent the same outcomes with novel antifungals as being currently observed with azoles.

## Agricultural practices driving AFR

Traditionally, azoles are applied annually; however, many farmers opt to routinely increase antifungal applications following heavy rainfall [163]. Aerial application of agricultural antifungals is associated with a ≥75 m drift area from the target site, and these fungicides are subsequently absorbed into the soil [164]. This is problematic as azoles in particular possess long half-lives, e.g. PPZ, which persists within the soil for ~300 days post-application [116,164]. However, after rainfall, the vast majority of the antifungals’ active ingredients leave the target site (i.e. the crop field) through agricultural run-off [164]. Hence, farmers will reapply antifungals to crops to ensure sufficient presence of active ingredients. This practice increases the selection pressure facing fungal pathogens [165]. Agricultural run-off can introduce sub-MIC levels of antifungals into aquatic environments, which may drive resistance, a phenomenon already documented in bacteria [116,166]. However, there are few studies which investigate this phenomenon in fungi [116]. In addition, the continual use of antifungals to protect crops from pathogens can inadvertently select against the crop’s own defence system [1]. These factors have led to programmes which advocate for farmers to move towards rotating antifungals and the application of higher doses at a more infrequent rate [116,167, 168].

Such strategies have resulted in the concept of ecological hotspots, where fungi grow in the presence of sub-MIC azole concentrations [126,169]. This in combination with other biotic and abiotic conditions is thought to create hotspots for resistance evolution [169]. Studies observing the growth of *A. fumigatus* in the presence of agricultural azoles noted this occurred often in composters and greenhouses [139]. A study investigating the potential of TBZ application to drive azole resistance within *A. fumigatus* showed there were significantly more resistant isolates from greenhouses compared to open soils [170]. This study argued this was due to the higher residual levels of TBZ within greenhouse soils [170]. Indeed, inhalation of azole-resistant *A. fumigatus* from a vegetable garden has been linked to azole-resistant *A. fumigatus* infection in an azole-naive patient [171].

Novel agricultural antifungals are being slowly introduced; however, it is crucial that current agricultural practices that drive the resistance are altered to limit the selection pressure against these novel antifungals. One such novel antifungal is mefentrifluconazole (MFT), which is utilized to control STB [172]. MFT belongs to a novel triazole subclass, the isopropanol triazoles, and has a flexible isopropanol unit [86,173]. This isopropanol unit allows MFT to be more tolerant of nonsynonymous substitutions within *CYP51,* which confers resistance to other azoles [86]. This is because MFT is structurally flexible, allowing it to effectively bind to Cyp51 even when the binding pocket has been structurally altered due to SNPs within *CYP51* [86,174]. MFT is therefore often more effective than other azoles in controlling *Z. tritici* [174].

## Other anthropogenic factors driving resistance

Climate change represents another driver of AFR (Fig. 2), however, unlike the other drivers, it is well-characterized as driving intrinsic AFR, which is unrelated to prior antifungal exposure [169]. The increasing temperatures force fungal pathogens to adapt, which can drive the emergence of novel human fungal pathogens [175,176]. Few fungal pathogens are able to grow at 37 °C, which is considered the thermal restriction zone that protects mammals from opportunistic fungal pathogens [65]. However, the warming world reduces the gap between ambient and hot temperatures, narrowing this thermal restriction zone [65]. For example, *C. auris* continues to predominantly colonize the skin rather than the gut microbiome, as it is much cooler [65,177]. The MDR nature of *C. auris* means it is predisposed to cause hospital outbreaks [11,177]. This intrinsic resistance is likely linked to the adaptation of *C. auris* to higher temperatures [169]. The thermal stress response in fungal pathogens is related to the alteration of the cell wall, specifically altering the ergosterol biosynthetic pathway, which changes the lipid composition within the cell membrane [169]. This can provide a mechanism for drug resistance as numerous antifungals target the fungal cell membrane [178].

The impact of climate change driving the emergence of AFR (Fig. 2) has been exemplified in the environmental fungus *Cryptococcus deneoformans* [179]*,* an opportunistic fungus which infects ~20% of European HIV+ patients [179,180]. Similar to many environmental fungi, *C. deneoformans* possesses a highly plastic genome, which allows it to rapidly adapt to environmental stresses, such as antifungal treatment [179,181,184]. In *Cryptococcus* species, ~5% of the genome comprises TEs, which can contribute to rapid genomic adaptation via gene rearrangements, inversions, deletions and translocations [179,185,187]. A recent study by Gusa *et al*. performed a forward-mutation assay to understand how *C. deneoformans* develop AFR [179]. They observed significantly increased transposon mobilization (predominantly TCN1 and TCN12) at 37 °C compared to 30 °C both *in vitro* and *in vivo* [179]. Increased TE mobilization resulted in increased insertional inactivation of both target genes, *URA3* and *URA5,* and therefore increased drug resistance [179]. The same phenomenon was observed under clinically relevant antifungal stress, with increased TE mobilization at elevated temperatures observed in numerous clinical and environmental strains [179]. These authors hypothesize that if increased TE mobilization at higher temperatures is observed in other non-pathogenic fungi, this may lead to adaptation of thermotolerance, which would allow these fungal species to become pathogenic [179]. Thermotolerance alone is typically not sufficient to confer full pathogenic potential. Other factors, such as the ability to evade the host immune system, produce virulence factors, and adapt to different environmental conditions also play significant roles in determining a fungal species' pathogenicity. Therefore, while thermotolerance is important, it must be considered alongside these other factors to fully understand a fungus’s capacity to cause disease.

If the same phenomenon occurs in crop pathogens, climate change would represent a major threat to food security. Therefore, the increased potential for the emergence of resistance and novel human fungal pathogens in response to climate change demonstrates how routine surveillance and species-level identification are crucial for effective therapeutic treatments [65].

## Future perspectives – One Health: an integrated approach to tackle AFR

Maintaining an effective antifungal arsenal is critical to protect both patient health and crop security. To achieve this, an integrated one-health approach that considers resistance drivers both in environmental and clinical settings is required. As such there are five main strategies which could be implemented to tackle increasing AFR: developing effective novel antifungals, implementing robust AFS, integrated disease management, improving diagnostics and improved surveillance.

Currently, there are 11 antifungals which are in clinical trials and 2 antifungals in production for agriculture [1]. Unfortunately, however, AFR emerges much more rapidly than the production of novel antifungals. This is especially true of novel antifungals, which have similar modes of action to existing antifungals. Therefore, novel antifungals which have unique modes of action are highly desirable. One such antifungal is T-2307, which is selectively transported into fungal cells, where it accumulates and alters the fungal mitochondrial membrane potential, which is crucial for respiration and generation of ATP. T-2307 is a broad-spectrum and is fungicidal against most major fungal pathogens, including *Candida*, *Aspergillus* and *Cryptococcus* spp. [188,191]. Another facet of the novel antifungal pipeline is drug repurposing. The homology between fungal and human genes can sometimes allow for the repurposing of existing therapeutics, which inadvertently possess antifungal activity. A prime example is AR-12, which was originally developed as a cancer therapeutic and progressed to phase I clinical trials. AR-12 displays antifungal activity against both yeasts and moulds; however, its mode of action remains unknown. Although identifying novel modes of action is desirable, novel and more effective versions of existing antifungals are in development [6]. However, if we do not modify the way antifungals are utilized within both the clinic and the environment, rates of resistance will continue to rise. This is of particular concern for the novel clinical antifungals OLF and fosmanogepix, which have structurally similar agricultural counterparts, IFQ and aminopyrifen [6].

Creating robust AFS programmes is one key strategy for preventing the emergence of resistance. AFS refers to introducing programmes which enforce the informed use of antifungals to prevent the unnecessary introduction of selective pressures to create effective stewardship programmes; the exact species of pathogen, the potential for resistance and alternatives to antifungals should be considered. Alongside preventing the emergence of resistance, preventing overreliance on antifungals is highly desirable. Clinically, there are numerous alternatives to antifungal treatment in development with varying degrees of feasibility. The adoptive transfer of activated antifungal immune cells has been shown to be effective against *Candida* spp., *Aspergillus* spp. and *Mucorales* infections *in vivo* by stimulating the host immune response to clear these infections [192]. Additionally, utilization of mAbs against invasive candidiasis infections has shown promise both alone and in combination with FLC, CSF and AMB, respectively [193].

There are also alternatives to agricultural antifungals in development. The most notable of which is utilizing genetically modified plant crops, which have increased resistance to fungal infections [194,195]. Although this represents an effective measure against fungal pathogens, many countries globally have banned GMOs, (genetically modified organisms), including some within the EU. Another more accepted alternative is utilizing RNA interference (RNAi) therapeutics to manipulate gene expression of the fungal pathogens, representing an effective approach. This can be performed via spray-induced gene silencing (SIGS), which introduces RNAi which when taken up by the fungal pathogen inhibits either growth or virulence [196]. The potential off-target effects of RNAi and the potential for resistance have yet to be elucidated. Crucially, alongside protecting crops, the losses within the field SIGS can also be utilized to protect crops against post-harvest losses, which can reduce yield by up to 25% [197,198].

Anti-infective and anti-biofilm agents offer promising alternatives to traditional antifungals by targeting pathogen virulence or disrupting biofilm formation, which enhances resistance. Anti-infective agents can inhibit specific fungal virulence factors or bolster host immune responses, reducing selective pressure for resistance. Anti-biofilm agents preventing biofilm formation or disrupting established biofilms could work to increase the efficacy of existing antifungal treatments. While these strategies hold great potential, challenges such as biofilm complexity must be addressed to ensure their effectiveness and safety. To create effective One Health strategies which tackle clinical and environmental drivers of resistance, all of the above suggestions must be considered to create integrated disease management strategies. Crucially, improved diagnostics and surveillance of fungal pathogens environmentally and clinically can allow for informed selection of antifungals and potential prevention strategies.

## Abbreviations

AFR: antifungal resistance
AFS: antifungal stewardship
AMB: amphotericin B
AMR: antimicrobial resistance
CSF: caspofungin
DFZ: difenoconazole
DHODH: dihydroorotate dehydrogenase
EPZ: epoxiconazole
FLC: fluconazole
ICU: intensive care unit
IFQ: ipflufenoquin
ISV: isavuconazole
ITC: itraconazole
mAbs: monoclonal antibodies
MDR: multidrug resistant
MFT: mefentrifluconazole
OLF: olorofim
POS: posaconazole
PPZ: propiconazole
PRO: prothioconazole
RNAi: RNA interference
SIGS: spray-induced gene silencing
STB: Septoria tritici blotch
TB: tuberculosis
TBZ: tebuconazole
TEs: transposable elements
TR_34_: 34 bp tandem repeat
VOR: voriconazole
WWTP: wastewater treatment plant
